# The Amazon Hope: A qualitative and quantitative assessment of a mobile clinic ship in the Peruvian Amazon

**DOI:** 10.1371/journal.pone.0196988

**Published:** 2018-06-21

**Authors:** Neha P. Limaye, Magaly M. Blas, Isaac E. Alva, Cesar P. Carcamo, Patricia J. García

**Affiliations:** 1 North Pacific Global Health Fellows, Fogarty and Kuskaya Fellowship, University of Washington, Seattle, Washington, United States of America; 2 Epidemiology, STD, HIV Research Unit, School of Public Health and Administration, Universidad Peruana Cayetano Heredia, Lima, Peru; University of Washington, UNITED STATES

## Abstract

The Loreto region of the Peruvian Amazon faces many obstacles to health care delivery. The majority of the population is river-bound and lives below the poverty line, with some of the worst health indicators in Peru. To overcome these barriers and fill a gap in health services, an NGO-based provider known as the Vine Trust has been providing care since 2001 via a mobile ship clinic called the Amazon Hope. This study presents an assessment of the Amazon Hope, first reporting health indicators of the program´s catchment area, services provided, and program utilization. It then describes perceptions of the program by community members and health workers, the program’s strengths and weaknesses in contributing to health service delivery, and provides recommendations addressing limitations. The qualitative analysis included 20 key informant interviews with community members and health service providers. In the quantitative analysis, 4,949 residents of the catchment area were surveyed about medical histories, experiences with the program, and suggestions for improvement. The survey showed poor indicators for reproductive health. The AH clinic was the main provider of health care among those surveyed. Community members reported satisfaction with the program’s quality of care, and health workers felt the program provided a unique and necessary service. However, community members requested prior notification and additional services, while health workers described misunderstandings in community-tailored care, and difficulties with continuity of care and coordination. Data show that the program has been successful in providing quality health care to a population but has room to improve in its health service delivery. Suggested improvements are provided based on participant suggestions and relevant literature. The study sheds light on the important role of mobile clinics in Peru, and the methodology can serve as a model for assessing the role of mobile clinics in other remote settings.

## Introduction

The residents of the Loreto, Peru’s largest Amazon region, face a difficult combination of barriers to health care delivery. The population is very sparsely distributed, with a population density of only 3.2 people per km^2^, versus 24.8 people per km^2^ nationally [1][2], and more than 50% of the population lives below the poverty level [3]. Citizens below the poverty line in Peru have guaranteed health insurance through a government program called *Seguro Integral de Salud* [4], but access to care is limited by geographic, financial, cultural, and health system barriers. With the Amazon river and tributaries crisscrossing the area, many communities are only accessible by boat, located hours away from each other and from any health service. Loreto also has one of the lowest physician- patient ratios; 4.4 per 10,000 as compared to the national average of 9.4 per 10,000, making it more difficult to access a Ministry of Health care provider [5]. Only 62.7% of the population has access to running water, and only 72.1% have drainage, creating an environment in which illness spreads more easily [3].

In this setting, it is not surprising that Loreto’s health indicators are some of the worst in Peru. Loreto has a high incidence of diarrheal disease in children 5 years old and younger (18.8 cases per 1000 habitants in 2015) and the third highest incidence in the country of pneumonia in children under 5 (191.6 per 10,000 children) [6][7]. The region also has the second highest infant mortality rate (30 deaths per 1000 births vs. a national average of 19 deaths per 1000 births), and a child mortality rate nearly twice the national average (40 per 1000 vs 23 per 1000) [8].

For hard-to-reach populations like that of Loreto, mobile clinics serve as a strategy to increase access to care. Mobile clinics are defined as “customized vehicles that travel to the heart of communities …They overcome barriers of time, money, and trust, and provide community-tailored care to vulnerable populations” [9]. The Amazon Hope (AH), a project supported by the UK based charity Vine Trust, began their mobile clinic services in Loreto in 2001, aiming to partner with the government to fill existing gaps in health care. The AH collaborates with the Peruvian Ministry of Health (MoH), and the Team of Comprehensive Health Care for Excluded and Remote Populations (AISPED) to provide medical and dental care to 160 remote Amazonian communities inaccessible by land. AH uses two medical ships that travel along six river basins within Loreto- Amazonas, Manatí, Tigre, Marañón, Ucayali and the Puinahua Chanel. These mobile clinics are staffed with a team of full-time Peruvian physicians and technicians and volunteer clinicians from different countries. The clinics are equipped with an operating room, dental services, pharmacy and treatment rooms, and serve over 100,000 persons annually. The mobile clinic ships visit the same communities every three months to provide preventive, medical and dental care, and carry out short-term specialist services to treat eye conditions such as cataracts and pterygiums.

MoH, AISPED, and AH work synergistically by covering different areas of healthcare- AISPED works in more remote areas, MoH provides vaccines, contraception, prenatal health, and vector-borne diseases, and AH covers general care needs. The program also occasionally works with Peru’s NAMRU—a NAMRU worker travels with the AH ship and provides urine tests, fecal analysis, and hematocrit measurements, and can test for malaria, leishmania, and filariasis.

The AH also provides additional preventive services including health talks during community visits about proper handwashing, water treatment, and use and handling of toothbrushes. The AH conducts initiatives like regular deworming of all patients and providing iron supplementation. The medical care includes general medicine consultations, and comprehensive antenatal care, including laboratory and pregnancy tests not provided by government posts. The dental care includes preventative care, basic restorative and extraction service [10].

Though the AH program has been running for over fourteen years, there has been limited investigation of the program´s acceptability and quality of services, or of its current role and limitations. Literature on the role of mobile clinics is limited in general [11], [12], and there is no published data regarding mobile clinics in Peru. This case study provides a formal assessment of the AH through the following steps. First, the study characterizes the population, the services provided, utilization by the population, and health indicators of the program’s catchment area. Second, the study uses qualitative and quantitative methods to understand community and health worker perceptions of the AH. Finally, using a framework based on the World Health Organization’s building blocks of health system strengthening [13], the study evaluates the program’s current function and offers potential solutions to its challenges. The study directly provides operations research data to the AH, and also serves as a model to determine the roles and contributions of mobile clinic services in other remote locations.

## Materials and methods

The design is a mixed methods study with a convergent parallel design, including qualitative and quantitative methods performed between September and December 2012. The protocol and instruments were approved by the Institutional Review Board (IRB) at Universidad Peruana Cayetano Heredia, in Lima, Peru (IRB00001014). The IRB waived the need for written informed consent from participants.

### Qualitative analysis

For the qualitative analysis, 20 key informant interviews were conducted by primary investigators Dr. Blas and Dr. Alva from September to November 2012. The interviews were conducted with key informants including personnel of AH and AH partners, a local NGO known as Amazon Promise, and with people living in communities served by the AH program. These personnel represented the main groups involved with AH, and interviewees were selected through purposive sampling, and sampling was complete after each of these major groups was represented. Each interviewee was approached face-to-face by the lead investigators, the study objectives were explained, and verbal informed consent was obtained prior to starting. No interviewee refused to participate in the study. The investigators had significant experience working in the area and research interest in public health in the Amazon, but had no specific connection to any interviewee or to the AH program. All interviews occurred in the workplace, only the participant and researcher were present, and all efforts were made to keep interviews private. Interviewees were asked in Spanish about common local health problems, previous experiences with the MoH and the AH Program, positive aspects of the program, and potential improvements, and the interviews were recorded via an audio recorder. Field notes were also taken, but not formally analyzed. The questions were based on an open interview guide that had been pilot tested by the investigators. Each interview lasted between 15–45 minutes.

Transcripts were made of each interview, but were not returned to participants. For analysis, an iterative approach was used to identify emerging concepts and thematic categories. Constant comparison was used during the coding and thematic analysis, which was done by two of the primary investigators through Microsoft Word. Transcripts were reviewed to identify illustrative quotes on the most common themes, and these quotes were translated to English. Participants were informed of the final results.

### Quantitative analysis

The community-based survey was conducted between October to December 2012

#### Sample selection

We calculated a sample size to determine the program coverage with respect to attentions and education using cluster sampling and communities as clusters. Assuming coverage of 50%, an alpha level of 5%, a design effect of 3, and error of 5% and a population size of 104, 530, a sample size of 1,149 households would need to be recruited. To increase the number of clusters, a maximum of 30 households per community was selected.

The sample was selected via a two-stage, random cluster sampling of the 117 communities reached regularly (every three months) by the AH program. In the first stage, 40 of the 117 communities were randomly selected. With the assistance of a community leader, in each community a map was drawn recording the location of all homes. Landmarks such as fields or churches were included, and houses were numbered and visited clockwise. A housing directory was created, including the home address, the name of the person who identified the house, and the total number of people living in the house. For the second stage 30 households per community were selected using a random housing table created from the housing directory. If the total number of inhabited homes in the community was equal to or less than 30, all houses were included in the survey.

#### Survey methodologies

The survey instrument had three sections. The first section contained questions for every household member regarding 1) demographic data and insurance information, 2) medical service utilization in terms of number of visits to AH and MoH and types of care received within the last year and 3) history of malaria in the past year, dengue, leishmania and hepatitis in the past five years, and lifetime history of diabetes, cancer, hypertension, tuberculosis, disability, drinking, and smoking, asking about diagnosis and treatment of these conditions. The second section contained questions for the female head of household asking more specific demographic information about time spent in the community, ethnic group, literacy and income. It then asked about experiences with the AH, preferred parts of the program, and things they would like to change. The third section had questions for all women of reproductive age focused on reproductive health including Pap smear history, pregnancy history, and contraception use. The questions regarding demographic characteristics and reproductive health were based on a previously population-based survey conducted in rural areas from the Amazon basin [14],[15]. The survey contained 92 questions, and was piloted prior to the study to ensure accurate interpretation of the questions.

Information was collected using EpiSurveyor Mobile (now called Magpi) [16], a mobile phone-based application for collecting data during field-based surveys. Information was sent to the database via SMS if there was cell phone signal in the communities, or sent later via Wi-Fi.

From each household selected for the study, team members approached the woman head of household. We approached women because in these communities, they spend more time at home than men and thus are more likely to know information about their family and be in contact with AH services. The purpose of the study was explained, and verbal informed consent was obtained. If the woman was younger than 15, she gave assent and her parent/legal guardian gave informed consent.

Each woman was then asked questions in the first section of the survey. If other members of the household were present, they were directly asked questions as well. Then, the female head of household completed the second portion, though if the female head of household was not present, the male head was surveyed. Finally, all women of reproductive age (15–49) within the household responded to the third section.

For the survey data, we used STATA (v11.0; Stata Corp., College Station, TX). To assess differences in proportions, we used Fisher’s exact test, and for comparison of means we used ttests. To account heterogeneity between populations and differences in sampling probabilities we present cluster-adjusted weighted prevalence unless otherwise indicated.

## Results

### Characteristics of the participants in qualitative interviews

All 20 people who were approached accepted to participate in the interviews. Key informant interviews were conducted with: 1) eight workers of the AH Program including physicians, nurses and crew, 2) three workers from the Loreto Regional Health of the MoH 3) a nurse from the MoH and a health promoter from a participating community, 4) a leader within the NGO Amazon Promise, 6) a leader within a partner organization of the AH, and 7) five people from the communities of Yucuruchi, Oran and San Roque.

### Characteristics of the participants in the survey

1194 households within 38 communities were randomly selected for participation and visited. Of them 153 were not occupied at the time of the visit, and in 5 the head of household rejected participation (participation rate: 99.6%). The remaining 1036 households were surveyed.

For the analysis we excluded two communities because they had less than two members who attended the Amazon Hope, precluding adjustment by cluster. We collected data from 4,949 community members (see Table 1 for full data). 2,598 (52.5%) of those within households were male and 2,351 (47.5%) were female, with an average of 4.8 persons per household. This population represents 0.49% of the total population of Loreto and 1.61% of the population at rural areas in Loreto [17] [18]. The average age of a community member was 23.8 years (range: 1 day to 97 years). In terms of literacy, 14.5% of women and 6.3% of men 18 years old and older were illiterate. The majority of households (71.0%) made 100 soles ($32 as of 2018) per month or less. Ninety percent had a National ID Number (NID). This number is necessary to access several governmental social services like JUNTOS (a cash transfer program for people in poverty), public health insurance and public education. Among the survey population, 54.8% of those 18 and older had reached only a primary education level, and 37.1% had no more than secondary education. The majority of the population (77.9%) had some sort of health insurance, most of them (97.2%) through the *Seguro Integral de Salud* (97.2%), the guaranteed government health insurance for those in poverty.

**Table 1:** Demographic data of household members who participated in the study.

Variable	Total Percent (95% CI)
**Age in years, mean (95% CI)**	23.8 (23.2–24.4)
**Age in strata**	
0–5	19.4 (18.4–20.5)
6–14	25.5 (24.3–26.7)
15–49	42.2 (41.0–43.3)
>49	12.9 (11.7–14.1)
**Gender**	
Male	52.5 (51.3–53.7)
Female	47.5 (46.3–48.7)
**Education level**^**a**^	
None	4.3 (3.4–5.2)
Primary education	54.8 (52.6–57.0)
Secondary education	37.1 (35.0–39.3)
Superior technical education	2.2 (1.4–3.0)
University education	1.6 (0.9–2.2)
**Has National Id Number**	90.0 (88.6–91.2)
**Has any type of insurance**	77.9 (75.7–80.1)
**Type of insurance**^**b**^	
Seguro Integral de Salud	97.2 (96.1–98.1)
EsSalud	2.8 (1.9–3.9)

^**a**^ Among participants 18 years old and older.

^**b**^ Armed forces was excluded because there was only one participant in this category.

### Current health indicators

#### Reproductive health

The surveys revealed low levels of reproductive health access and utilization. 13.6% participants ever having a Pap test, reporting mainly the lack of Pap smear campaigns (61.9%) and fear of getting hurt (29.4%, Table 2) as reasons for not getting tested. The population had an average age of first pregnancy of 17.7 years (95% CI: 17.4–17.9). In terms of family planning, the main method reported was periodic abstinence (41.4%). 71.0% of pregnant women surveyed reported their current pregnancy as unwanted.

**Table 2:** Sexual and reproductive health of 958 women 15–49 years of age who live in communities visited by the Amazon Hope program.

	Female Percent (95% CI)
Ever had a Pap test	13.6 (11.3–16.0)
Number of pregnancies among women who were ever pregnant, mean (95% CI)	4.2 (4.0–4.3)
Number of children currently alive among women who were ever pregnant, mean (95% CI)	3.8 (3.6–4.0)
Pregnancies who ended up in a live birth	95.2 (94.3–96.1)
Percentage of home deliveries	80.2 (77.9–82.5)
Currently pregnant	6.8 (5.0–8.6)
Use of a family planning method among 702 women with a partner	81.9 (78.9–84.9)
Tubal ligation	1.2 (0.3–2.1)
Breastfeeding	3.6 (2.1–5.1)
Periodic abstinence	41.4 (37.1–45.7)
Contraceptive pills	21.8 (18.2–25.3)
Injectable contraceptive	31.4 (27.4–35.4)
Condoms	0.6 (0.0–1.3)

### Services provided by Amazon Hope

In the survey, 44.7% of participants reported a family member attending health talks offered by AH, and 57.1% of children were reported to receive ferrous sulfate from AH within the last year.

The survey found that the majority of household members who had received care from AH received general medical care (90.3%), but some also received dental care (12.1%) and a few had surgical care (0.21% receiving cataract surgeries).

The services provided by the AH program were also assessed during the study’s qualitative analysis. Interviews with AH and MoH personnel and community members revealed that the AH is the only provider in the area with specialty services, such as yearly ophthalmological care. The AH is also the only provider treating chronic diseases such as diabetes, epilepsy and hypertension, and can provide services regardless of insurance status or possession of documentation. Comments included:

- "*For me it is a benefit that the AH gives medication for hypertension and diabetes because as I said, this is a health post and we don't have medicines for that, we don't do diabetes screening""—Nurse from MoH Health Post*.
- *“The ship treats patients very well*. *The doctor gave me a prescription for my vision*, *drops and capsules*, *and I felt better with these medicines”–Community member*
- *“What I like about working at Amazon Hope is that we reach the entire population without exception*, *whether they have a national id document*, *a name*, *whether they live near or far away*, *or are Catholics*, *atheists*, *that's what I like*. *We reach people who are most in need*, *who sometimes tell us ‘Here they do not come from the health center*, *the brigades don’t come*.*’” (Worker from the AH program)*

Starting in 2010, the program conducted a study to improve anemia. Per their study the prevalence of anemia in children under five was 89%, with the highest prevalence in children 6–11 months old (96.9%). After a yearlong ferrous sulfate intervention targeting all children 6 to 59 months old who accessed care from the program, the prevalence was reduced to 20% in 2011 [19]. Interviewees indicated a desire for more sanitation interventions, given that most communities lack access to clean water, and many community members drink directly from the river. Health workers suggested distribution of a simple home filtration system as one potential solution.

### Comparison of participants who did and did not seek care at the Amazon Hope Program

We compared the characteristics of community members who did and did not seek care at the AH (Table 3). We found that participants who sought care at the MoH, those with any type of health insurance, and among them those with SIS were more likely to seek care at the AH. We found that male participants, and those aged 15 and older were less likely to seek care at the program.

**Table 3:** Demographic data of households members who did and did not seek care at the AH program.

Variable	Sought health care at the Amazon Program within the last 12 months		Total	Prevalence Ratio (95% CI)
	**Yes** **(N = 2,883; 58.5%)** **N (%)**	**No** **(N = 2,042; 41.5%)** **N (%)**		
**Age in strata**				
0–5	21.5 (20.1–22.9)	16.7 (15.0–18.4)	19.4 (18.4–20.5)	Ref
6–14	29.6 (27.9–31.2)	20.1 (18.0–22.1)	25.5 (24.3–26.7)	1.03 (0.99–1.08)
15–49	37.3 (35.8–38.8)	48.7 (46.5–50.8)	42.2 (41.0–43.3)	0.88 (0.85–0.92)
>49	11.7 (10.2–13.1)	14.5 (12.7–16.4)	12.9 (11.7–14.1)	0.89 (0.84–0.94)
**Gender**				
Female	56.6 (54.8–58.3)	35.5 (33.4–37.5)	47.5 (46.3–48.7)	Ref
Male	43.4 (41.7–45.2)	64.5 (62.5–66.6)	52.5 (51.3–53.7)	0.81 (0.79–0.84)
**Has National Identification Number**	92.8 (91.4–94.1)	86.0 (83.8–88.3)	90.0 (88.6–91.2)	1.02 (0.99–1.05)
**Has any type of insurance**	84.0 (81.9–86.1)	69.8 (66.2–73.4)	77.9 (75.7–80.1)	1.22 (1.16–1.29)
**Received Care from Ministry of Health**	65.5 (63.0–67.9)	40.2 (37.0–43.5)	54.6 (52.4–56.8)	1.28 (1.24–1.33)
**Type of insurance**^**a**^				
EsSalud	1.9 (1.1–3.2)	4.2 (2.7–6.4)	2.8 (1.9–3.9)	Ref
Seguro Integral de Salud	98.1 (96.8–98.9)	95.9 (93.7–97.3)	97.2 (96.1–98.1)	1.22 (1.04–1.43)

^**a**^ Armed forces was excluded because there was only one participant in this category.

In terms of health care received at the MoH, overall, 54.6% (95% CI: 52.4–56.8) received health care within the last 12 months, 44.3% were male and 55.7% were female. Of note, 34.6% of those seeking care from AH in the past year did not receive any care from the MoH within that time period.

**Fig 1:**
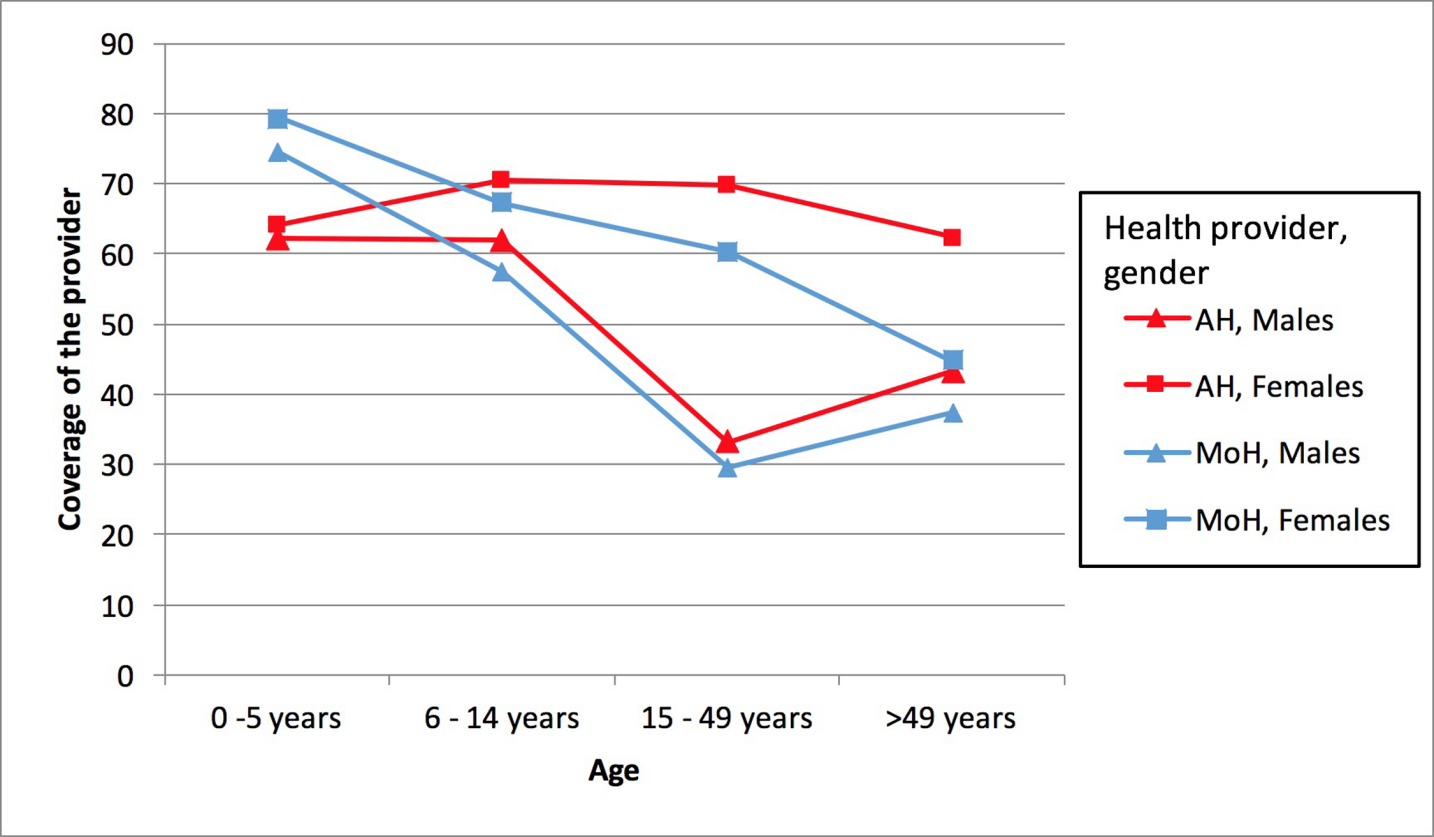
Healthcare provided by Amazon Hope and Ministry of Health, divided by age group and gender. AH: Amazon Hope, MoH: Ministry of Health.

Data was collected on how many times each household member sought care from AH and the MoH within the past year, with health care defined as medical, surgical, dental, pharmacy or laboratory services, or vaccinations. Fig 1 shows the proportion of household members from each age strata who received care at the AH and the MoH within the last year. The group of 0–14 years olds were the largest proportion of health seekers, while males age 15–49 were the group least likely to seek care from the AH. The main reported reasons for not seeking care were that the participant did not need care (40.8%), the mobile clinic came when the participant was away (31.0%), and that the clinic was docked far from the patient’s home (7.9%). Compared to MoH, AH had more coverage across all age and gender groups except for children under 5 (Fig 1).

### Perceptions of the Amazon Hope Program

Community perceptions were collected during both the quantitative and qualitative analysis. Heads of household were surveyed about what they liked most of the program, which aspects of the program could be improved and which additional services they would like to receive, with options provided based on the findings from the qualitative analysis. Health personnel perceptions were collected during key informant interviews. The perceptions of the AH program are below, separated by themes.

### Coverage and accessibility

Both community participants and health personnel noted that the AH increases accessibility to services and broadens coverage. Community Participants indicated that the AH has been the only mobile clinic that has visited their communities:

“*The little boat cares for us very well*. *It is the only [care provider] that comes here*. *Before*, *the navy ship would come*, *but there were no doctors on it*.*”-Community member*

Health personnel also noted the value added of the clinic in terms of increasing coverage.

- "*We are a viable alternative for people who do not have access to health services or who have very little access…at least people know every three months they can be treated by a doctor*, *by a dentist*, *they can access medicines""- AH personnel*
- *“We usually go to communities where there is no health post and if there is a health post*, *sometimes they only have a nurse technician who tells us "Doctor*, *I don’t even have Paracetamol” They are satisfied that we went and provided health care to the community*.*” (Worker from the AH Program)*

In terms of access to medication in the survey, the community responded that they appreciated the medication provided (63.7%) but also requested a larger variety of medicines (42.0%).

However, key informants interviewed also expressed some frustration with lack of communication with the program, which made accessing the clinic more difficult. For example, one community member noted that the mobile clinic would sometimes suddenly appear without warning, while he was out in his farm field. They suggested earlier notification from the program, perhaps via phone calls or radio messages.

### Quality and comprehensiveness of care

In the quantitative survey, community participants noted they most appreciated the quality of care provided by the program (82.5%). Female participants were more likely to indicate liking that providers were truly concerned about them (42.4% of females versus 24.5% of males, p = 0.01).

Interviews with community members overall revealed satisfaction with care provided by the program, and comments that it had higher quality care than the health post:

- *“The health post does not have the necessary equipment to work*, *in the post they do not cure us*. *When the little ship comes it always gives solutions that the health post does not give us*. *The little ship gives us medicines*, *vitamins and free purgatives*.*"- Community member*

However, in terms of service provision, nearly all community participants wanted additional services (97.5%). Participants most often requested blood tests (72.7%), ultrasound services (70.2%), and X-rays (64.3%). They also requested surgeries (30.8%), contraceptives (13.5%), and small local pharmacies (7.5%).

Health personnel also noted a lack of certain critical services during interviews. AH workers cited several cases of patients requesting reproductive and family planning services at the AH clinic but not receiving them. They also noted that the lack of skin biopsies tools is limiting—all dermatologic cases must be referred to Iquitos, and many patients cannot follow up their referrals. Finally, they noted that the clinic’s ultrasound often malfunctions, and that there is no X-ray equipment.

### Person-Centeredness

During interviews, health workers noted a number of challenges with communication between the AH personnel and the population they are serving. AH personnel noted that patients struggle with comprehending and following their prescriptions. Patients have difficulty understanding the written prescriptions, and there are instances of miscommunication with foreign doctors volunteering with the program. In the community, medicines can be misused or lost due to lack of storage space and flooding. There were multiple anecdotes of patients misusing medications or giving them to family members. Examples include:

- *“The patients sometimes do not understand the explanation we give them on how to take their medicines*. *Others do not know how to read*. *You have to explain to them very well and sometimes we are not sure if they understood everything and if they are going to take their medicines as we indicated*.*”- AH personnel*
- *"After the boat leaves the community*, *patients come and tell me 'they gave me this prescription but I don't understand*.*"- Local community health worker*

### Continuity of care

As the AH only arrives once every three months, continuity of care is a challenge. Community survey participants most often requested more frequent mobile clinic visits (49.8%).

Health personnel also touched on lack of patient follow-up. The program has no permanent medical records, making it difficult to follow patients over time or assess change. Health personnel suggested implementing an electronic medical record as the ship itself has no space to store paper records.

- *In the adult population we have greatly improved their access to health services*, *but I cannot objectively say that we decreased the prevalence or incidence of diseases*. *We have not conducted studies*.*"- AH personnel*

We don't know what medicines have been prescribed to them, what treatments they've had or if they had any problem- it's like the patient is coming for the first time each time""-AH personnel.

### Coordination between health care providers

A common theme noted in health personnel interviews was the strong relationship between the AH and the regional government. The AH coordinates with the regional health office to schedule community visits. Though the relationship with regional government is strong, on the ground, collaboration is more problematic. First, personnel noted difficulties with transitions of care—malaria and leishmaniasis are diagnosed by the NAMRU worker while on the AH ship, but are treated at government health posts; many patients are lost in transition. Second, although AH can provide comprehensive prenatal care, local government health workers prefer to provide care to fill out the official government prenatal care card. A solution suggested for this was to have a MoH midwife and nurse travel with the AH ship and provide care. Third, despite the AH program having better access to vaccinate remote populations, government workers prefer to vaccinate by themselves as they receive payments to conduct these visits. Additionally, at the time of the interviews minimal coordination between the program, local health promoters, and other NGOs was noted. Heath workers suggested including coordination with local teams.

## Discussion

The AH provides health care in a unique setting, serving a particularly hard-to-access region only reachable via ship. There are very few other mobile ship clinics in the world, and the only one described in the literature serves a similar role to AH, providing primary care services in a highly remote area [20]. There is limited data on mobile clinics in general, and few systematic reviews of their role within health systems, making it difficult to directly compare the AH to other clinics in terms of strengths and weaknesses. However, from the limited review papers [9,11,12], it is clear that mobile clinics are a common approach to health system strengthening, and the AH’s role can be assessed by how it contributes to the health system.

The latest World Health Organization guidelines present a methodology to monitor health system strengthening through the six building blocks of a health system: access to essential medicines, health workforce, health service delivery, health information systems, financing, and leadership/governance [13]. The AH contributes to Peru’s health system within multiple of these building blocks. The survey data and interviews show that the AH increases access to essential medicines. Multiple interviewees noted the ship having medications that local government clinics do not, such as medications for epilepsy, diabetes, and hypertension. AH also contributes to the health workforce through the volunteer and local providers that work together on the clinic ship. The following analysis, however, focuses on the AH’s role within the third building block, health service delivery.

### Service delivery

Health service delivery is a main area in which mobile clinics can contribute to health systems. Per the WHO framework, strengthening service delivery involves optimizing eight key characteristics of good service delivery. Below is a breakdown and discussion the Amazon Hope’s strengths and weaknesses within each of these characteristics (see Table 4), which align closely with the perceptions of community members and health personnel detailed in the data above. Solutions to the challenges are offered based on participant comments and the literature.

**Table 4:** Amazon Hope’s performance within the WHO components of service delivery.

Characteristic of good service delivery	Definition	AH Strengths	AH Weaknesses
**Coverage**	*Service delivery is designed so that all people in target population are covered*, *including sick and health*, *all income groups and social groups*.	•Reaches riverine population in extreme poverty •Higher rate of coverage than MoH across majority of age groups, and provides coverage to areas where MoH does not	
**Accessibility**	*Services are directly and permanently accessible with no undue barriers*, *and health services are close to the people*, *with a routine point of entry to the service network at the primary care level*	•Majority of survey participants could access AH care.	•Some community members do not receive information about AH schedule, miss chances to access care
**Quality**	*Health services are effective*, *safe*, *centered on patient’s needs and given in a timely fashion*.	•Participants most appreciated quality of care of AH •Females particularly felt AH providers were truly concerned about them.	
**Comprehensiveness**	*Provides comprehensive health service appropriate to needs of target population*, *including preventative*, *curative*, *palliative and rehabilitative services*, *and health promotion activities*.	•AH provides many preventative and curative services.	•Reproductive health care and prevention programs are limited in scope •Technological services (X-rays, ultrasounds, biopsies) are not consistently available.
**Person-centeredness**	*Services are organized around the person*, *not the disease*. *Users perceive health services to be responsive and acceptable to them*, *target population participates in design and assessment*.		•Patients misunderstand prescriptions and care plans due to non-tailored communication
**Continuity**	*Individuals are provided with continuity of care across the network of services*, *health conditions*, *levels of care*, *and over the life cycle*.	•AH attempts to coordinate follow-up with specialty providers.	-Inherent difficulty providing continuity of care when AH visits every 3 months.
**Coordination**	*Local area health service networks are coordinated across types of provider and level of service*. *Primary care provider facilitates route*. *Coordination exists between other sectors and community partners*.	•Strong connections between AH and regional government.	•Room to strengthen connections between AH, community health workers and local government services
**Accountability and efficiency**	*Health services are well managed with minimum wastage*. *Managers are held accountable for overall performance and results*, *with regular assessments*.		•Data collection to assess AH performance very limited.

## Coverage

Successful healthcare delivery requires good coverage, ensuring that all people within the target population, in this case the residents of Loreto, can receive care. Overall, interview and survey data show the AH program is able to reach a population in extreme poverty with multiple determinants of poor health, including limited access to reproductive health services. The Amazon Hope reaches greater than 50% of the surveyed population across all age groups barring males over 15 years old. Overall, the program has a higher rate of coverage than the MoH across all age groups except for children under 5 years old. Children under 5 likely have more contact with the MoH because the MoH conducts children’s’ vaccination campaigns, and vaccinations were included in measures of coverage. Importantly, the data shows that AH provided coverage to a large proportion (34.6%) of participants who did not receive any care from the MoH, demonstrating that AH helps improve healthcare coverage in the area.

### Accessibility

The AH provides potential healthcare coverage to a large population, but it is also essential that patients can actually access this care. Per the WHO, accessibility means that services are “directly and permanently accessible with no undue barriers…close to the people, with routine point of entry to the service network.” Our survey showed that the majority of participants are able to access care via the AH program, and utilize the program more frequently than other care providers. The nature of AH as a mobile clinic, being able to reach smaller riverine communities by ship, increases healthcare access. However, interviewees requested better notification of the mobile clinic’s schedule, and the data shows that young men were least likely to visit the mobile clinic.

As one interviewee suggested, local community health workers and clinics could be notified a few days prior to the mobile clinic’s arrival, enabling time for community preparation. Young males are least likely to seek care at the AH, Interviewees noted that young men often miss the clinic visits because they are often out working in the fields. Young males seeking less care is a common pattern seen across many countries, and has been attributed to multiple sociocultural factors including masculinity constructs, man-unfriendly health care, delays in help-seeking, and an increased likelihood to seek help from partners and friends rather than from health care providers [21][22]. From the literature, interventions that have been successful in improving male health-seeking behavior utilize male-specific language, and are designed in conjunction with local male community members [23]. Given their lower rates of access, the AH could specifically target young men and design a program tailored to inspire them to seek care.

### Quality and comprehensiveness of care

Next, to contribute to health services delivery, it is critical that the healthcare being accessed on the AH is comprehensive, high quality care. High quality health services are effective, safe, centered on patient’s needs, and given in a timely fashion. Comprehensive services include preventative, curative, palliative and rehabilitative services and health promotion activities. Interviewees and survey participants described satisfaction with the care provided by the AH clinic. Their main request was for the mobile clinic to come more frequently, indicating a high opinion of the program’s care. However, several limitations exist in AH’s scope of care. As seen in Table 2, reproductive health care access is limited. The average age of pregnancy is significantly lower than the national average (17.7 vs 21.9) [24], there is a high percentage of home deliveries (80.2%) and the rate of pap smears was markedly lower than the rate of 31–43% described in other areas of Peru [25],[26]. Finally, health workers and community members strongly requested more technological services- 99.3% of those surveyed requested X-rays, and health workers noted the malfunctioning ultrasound multiple times.

To address these limitations, the mobile clinic should first consider offering family planning and women´s health services. Mobile pap smear clinics and more recently HPV self-testing have proven to be successful in improving rates of cervical cancer screening in other regions of Peru [27] [28], and the AH could have a similar impact, especially given the extremely low baseline rate of cervical cancer screening. Using visual inspection with acetic acid would work particularly well in this low follow-up, low resource setting [29]. 13.6% of patients requested contraception services at the AH clinic, indicating there would be utilization if this service were offered. At the time of this study the only provider of contraception was the MoH.

Third, building capacity for dermatology screenings, consistent ultrasounds, and x-rays would greatly enhance the care provided. In dermatology, studies have shown success using telemedicine to increase access to specialty care in highly remote locations [30] [31].The AH could use telemedicine to communicate with dermatologists and provide direct care in the mobile clinic.

In terms of prevention, The AH has had a series of successful prevention programs, including the described anemia program, a deworming program, and health talks in communities, which were very popular per the survey. They also perform comprehensive antenatal care and pregnancy tests as well as periodic ophthalmologic visits. However, the AH is not currently addressing sanitation and water safety issues.

As suggested by the health workers, the AH could partner with schools, community-based organizations and NGOs to have more consistent education programming, rather than once every three months. To improve sanitation, the AH could work with government health workers and community members to design a collaborative water program. Point of use water chlorination is an affordable method which has been shown to reduce the risk of child diarrhea and water contamination in multiple developing countries [32]. Improved sanitation could directly affect Loreto’s elevated infant mortality, as one of the most common causes of infant mortality worldwide is diarrhea from poor sanitary conditions. Simple filtration interventions have been shown to make significant impacts on diarrhea prevalence. As the AH already has built trust in the communities, it would be a feasible intervention to implement [33].

### Person-centeredness

The AH is providing a high quality of care and is fairly comprehensive in its services, but is this care tailored to the specific population? Per the WHO, services should be organized around the person, not the disease, and there should be participation from the target population in service delivery design. As the heath workers noted, the AH program has multiple challenges in meeting this expectation. More community-tailored approaches to facilitate prescription understanding could be used, such as:

Prescriptions with diagrams and pictures: As the majority of the population has primary level education, medical providers need to take extra care with prescriptions. Prior studies in low literacy populations show the success of using pictures and diagrams to improve adherence [34]. For example, prescribers could use symbols of the sun and moon to indicate medication schedules. They could also assign a number for each medication, so that patients would only have to recognize the number on each box rather than read the name [31].

Teach-back method: This method is also used in low literacy populations and has been shown to improve patient comprehension after one-time patient visits [35]. Before leaving the clinic, the patient is required to explain their prescription and instructions to a health provider. If they seem unsure, the instructions can be re-explained. If the patient was treated by a foreign clinician, a native speaker can ensure the patient understood the translation.

Prescriptions used only for intended purpose: When prescribing, AH personnel should emphasize not to share medicines, not to self-medicate, and to keep medications out of reach of children, as these were all noted to be significant issues.

### Continuity

The above discusses the health services provided during each AH visit, but continuity of care over time is also very important for service delivery. It should be organized to give patients continuity of care across services and health conditions. The AH inherently has difficulty with continuity of care as it is a mobile clinic that only comes into each community every three months. This was noted frequently as a difficulty by the health personnel interviewed. It was also noted that patients with malaria and dermatology referrals are frequently lost to follow-up. Some potential solutions to improve continuity of care are as follows:

Increase Treatment On Board: Providing treatment on board for those diseases which are known to be common (e.g. malaria) would streamline the process of diagnosing and treating the disease and minimize loss to follow-up.

Electronic Medical Record: The AH could consider simple data collection methods such as data entry via mobile device. Maintaining an electronic medical record would be particularly beneficial for tracking patients. Right now every patient visit is essentially a new patient visit, which makes it difficult to treat any chronic issue. Also, electronic data could be shared with the MoH, vastly improving patient management between different care providers. The AH program could also provide inexpensive phones to community agents, enabling the mobile clinic to send reminders and communicate with patients via the community agent.

Local medical cabinets: The mobile clinic could create small local pharmacies within the communities staffed by trained community health workers to provide follow-up medications between mobile clinic visits. This was indicated as a desired service by 7.5% of the heads of household surveyed.

### Coordination

All of the above characteristics focus on the care the AH itself is providing, but it is also essential for the AH network to be coordinating actively with other types of providers, and to facilitate the route to other types of service needed. This is a key component of health service delivery within a larger system. As health personnel noted, the AH has a strong connection to the regional government, but could improve relationships at the ground level. There are a variety of methods to boost local relationships. First, the AH could use existing or new community health workers to assist with continuity of care. Community health agents are a proven method of improving access, utilization, and understanding of healthcare across the world [36], [37]. As suggested above, agents could notify the community of the AH clinic’s visit and manage local medicine cabinets. Agents could also work directly with the AH to ensure patient comprehension and follow up for referrals, as many already were doing informally.

To improve coordination with local government services, MoH personnel could travel with the AH ship. One suggestion was for a MoH midwife and nurse to provide prenatal care, contraception, and vaccinations on the AH ship. Given that space and budget on the ship is limited, another viable option is telemedicine. Some of the AH’s catchment area has cell phone signal, thus it would be feasible for AH members to communicate with government workers, and to collaborate in patient care decisions.

In terms of integrating with other services, the AH program could use the government’s PIAS program mentioned above as a model, combining its medical services with the opportunity to provide other social services. If the AH program can strengthen ties with local collaborators, it could provide a combination of services like PIAS in one location.

### Accountability/Efficiency

Finally, per the WHO, health services should be able to assess their performance for accountability. Overall, as described above the AH does not collect data or keep medical records, and beyond the anemia study there are no impact measurements. Having a records system would greatly improve patient follow-up and create a rich data base for a true impact evaluation.

### Progress in AH services from 2012 through 2018

Since data was collected in 2012, by 2018 and as a result of the findings from this study, the AH has already implemented several of the suggested changes, including: 1) revised strategies for notification of mobile clinic visits, 2) improved the prescription system using the sun and moon design and prescription explanation by both the doctor and the pharmacist, 3) signed agreement with the Regional Government of Loreto and the MoH that are now assisting with petroleum, medicines, health care personnel (physician, nurse, professional midwife, dentist, laboratory technician) to provide care to people in the area, 4) increased the stock of available medicines (from 96 to 127 items), 5) increased collaboration with AISPED and the district health center to provide contraceptive, malaria treatment and pap smear services, 6) collaboration with Mamas del Rio (Mama River) program, connecting community agents with the AH through cellphones, 7) current development of an electronic medical record system to be piloted in 2018, and 8) new ultrasound equipment available onboard the ship. Additionally, the Amazon Hope deployed on 2018 in Peru a new Medical Ship called Forth Hope that will expand the Amazon Hope services to other basins. Also, in 2015 the Amazon Hope deployed a medical ship on Lake Victoria, Tanzania, which focuses on providing primary healthcare services, particularly around HIV/AIDS, to the islands of the Kagera region. As the AH continues to make more suggested changes, it will further improve health service delivery in the region.

### Limitations

The study is limited by several factors. It is a cross-sectional study and therefore cannot be used to determine causality. The study did not include any economic or cost effectiveness analysis (maintenance and fuel costs of the operation of the AH are considerable), these data would have provided an important perspective on the utility of the AH mobile clinic’s activities. Cost analysis is also essential for scaling up or putting any suggested interventions or improvements into action. Also, the number of interviews in the qualitative data set was limited. One improvement or possible future study would be conducting exit interviews with patients after their mobile clinic visit. This would provide immediate perspectives on the AH, rather than surveying participants possibly months after their last interaction with the AH.

## Conclusions

In conclusion, at the time of data collection, the AH was a well-liked service that was helping strengthen the health system of Peru by providing several components of health service delivery, improving coverage, and complementing services provided by the MoH. Several areas for improvement were identified as well, particularly within improving coordination, person-centeredness of care, and comprehensiveness of services. Practical actionable solutions were offered, and any of these changes have already been implemented. The next steps for this study will be working directly with the AH and communities to determine which further recommendations are most feasible. It is also essential to continue operations research as changes are implemented. The study clearly shows that the Amazon Hope plays a significant role in health care provision in Loreto, and should inspire further research on the role that mobile clinics play within health systems. The methodology used by this study can be combined with a cost analysis and could serve as a starting point in assessing and improving other mobile clinic services in remote locations.

## Supporting information

S1 Photo The Amazon Hope, pictured traveling down one of the tributaries of the Amazon river in parinari, Loreto, Peru. This mobile clinic ship provides services to many indigenous communities in Loreto that are only accessible via river.
(JPG)
