# Supplementary figures and images for: The Amazon Hope: A qualitative and quantitative assessment of a mobile clinic ship in the Peruvian Amazon
Source: PLoS One. 2018 Jun 21;13(6):e0196988. doi: 10.1371/journal.pone.0196988 (PMC6013175; doi:10.1371/journal.pone.0196988)

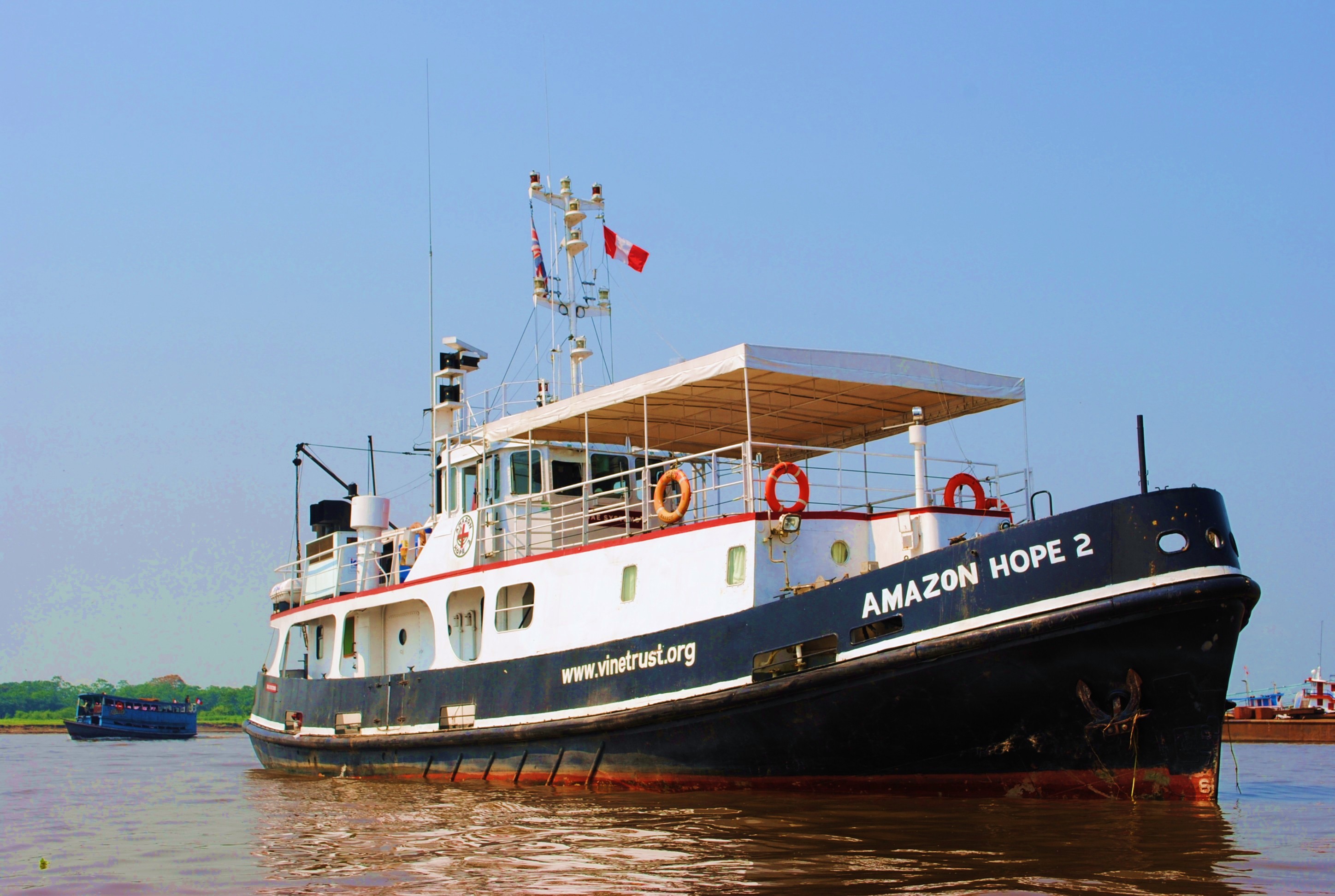

Supplement: S1 Photo — This mobile clinic ship provides services to many indigenous communities in Loreto that are only accessible via river. (JPG) [file pone.0196988.s001.JPG]
